# Comparison of Oral Recombinant Erythropoietin and Subcutaneous Recombinant Erythropoietin in Prevention of Anemia of Prematurity

**Published:** 2012-03-01

**Authors:** R Saeidi, A Banihashem, M Hammoud, M Gholami

**Affiliations:** 1 Department of Pediatrics, Mashhad University of Medical Sciences, Mashhad, Iran; 2 Department of Midwifery, Islamic Azad University, Neyshabur Branch, Neyshabur, Iran

**Keywords:** Anemia, Prematurity, Recombinant erythropoietin, Newborn

## Abstract

**Background:**

Premature neonates are at risk for severe anemia and erythropoietin is the most important hormone in erythropoiesis. The aim of this study was to evaluate the influence of oral recombinant human erythropoietin (rhEPO) in proving erythropoiesis in neonates.

**Methods:**

This was a randomized clinical trial study. Thirty neonates were enrolled from September 2007 to September 2008. The first group received oral rhEPO and Fe and the second, subcutaneous rhEPO and Fe. The patients’ Hb, HCT and the need to blood transfusion were recorded. We included all infants with gestational age <34 weeks, birth weight <1500 gr, without respiratory distress (O2 Saturation> 85%, FiO2 of 30%), full feeding tolerance so that oral Fe can be administrated.

**Results:**

In first group (oral=PO), 65% of neonates were female and 35% were male, mean weight was 1140 g and mean GA was 32.6 weeks. In the second group (subcutaneous=SC), 42% were female and 58% were male. The mean weight was 1245 g and mean GA was 31.2 weeks and this was not statistically significant. In the first group, the mean Hb and HCT were 9.7±1.9 and 29.6±5.9 g/dl. In the second group, the figures were 12.5±1.7 and 38.8±5.1 which were statistically significant. There was no difference in the weight gain between two groups. In the first group, 3 neonates (20%) and in the second one, 1 neonate (15%) needed blood transfusion.

**Conclusions:**

rhEPO administration either PO or SC could prevent anemia of prematurity but SC rout was more effective.

## Introduction

Anemia in neonates is defined as more than 2 SD under the mean value of the amount of hemoglobin or HCT for a given age.[1] Anemia of prematurity starts at third or fourth week after birth and the amount of Hb may decrease to 7 g/dl.[2] It sometimes has clinical manifestations like pallor, apnea, no weight gain, decrease in activity, tachypnea, tachycardia and nutritional problems that necessitates blood transfusion.[1][2]

On the other hand, recombinant human EPO (rhEPO) is the major factor in erythropoiesis in the fetal and neonatal period as well as in puberty by inhibition of apoptosis of progenitors in erythroid lineage and provoking their proliferation and differentiation to normoblasts.[3] Another benefit of rhEPO is the protective effect against ischemic encephalopathy in neonates.[4]

The use of rhEPO for treatment of anemia goes back to1985.[5] Serum level of rhEPO in premature neonates is lower than mature neonates and after birth its level decreases even more.[6] This is one cause of blood transfusion in premature infants.[2] It is known that rhEPO does not cross the placenta. Jull et al. has observed that rhEPO found in mother's milk is produced by mammary glands and is secreted into milk.[5] It has been observed that the level of rhEPO is added to mother's milk as standard formula milk under conditions similar to that of the stomach or intestine remaining constant, but rhEPO added to 5% dextrose serum, undergoes digestion.[7]

Erythropoietin has many properties and its receptors are found on liver, endothelial, smooth muscle, placenta, brain and testis cells as well as on cardiomyocytes and erythrocytes.

We know that blood brain barrier and blood testis barrier prevent the passage of this hormone; it is possible that rhEPO is synthesized in these organs for reasons other than erythropoises.[8][9][10][11] Since 1985, when rhEPO was available in market, it was used in the prevention and the treatment of anemia of prematurity. It has some side effects that were reported in neonates.[10]

According to Ahmadpoor et al. study, rhEPO can decrease incidence of anemia of prematurity and its complications. In Ballin’s study,[6] premature neonates received only Fe drops and 6 other neonates were administered Fe drops and rhEPO.

In the rhEPO group, no complication was reported and serum level of rhEPO and reticulocyte number increased significantly. Ferritin level decreased, but the need for transfusion did not decrease.[1] Although it has been approved that rhEPO plays a role in the prevention of prematurity anemia, but in most clinical trials, rhEPO was administrated intravenously or subcutaneously (IV, SQ). Few studies were carried out to determine the effectiveness of rhEPO in prevention of prematurity anemia and even they had controversial results.

In this study, we compared the effect of oral rhEPO with subcutaneous rhEPO and investigate its effectiveness in prevention of anemia of prematurity. We have evaluated a simple and more suitable way for prevention of anemia of prematurity.

## Materials and Methods

This study is a randomized clinical trial, in which 30 neonates having the qualifications of the study, were studied from first September 2007 to 1st September 2008 in Quaem Hospital in Mashhad (Iran). During a period of one year, 30 premature neonates, having an age of less than 34 weeks and a weight under 1.5 kg and were hospitalized in the hospital were randomly divided into two groups.

The first group was given oral rhEPO and oral Fe (rhEPO: 400 u/kg per weeks, Fe: 4 mg/kg/day). RhEPO was given during the first week after birth and at the beginning of the trials as well as in the 2nd and 4th week, the level of HCT, Hb and reticulocytes was checked.

Also weight gain, the need to blood transfusion and other information related to this study were recorded by a researcher daily. Other information was recorded using a questionnaire. The inclusion criteria in the study comprised all infants with gestational age less than 34 weeks, birth weight less than 1500 gr, absence of respiratory distress (O2 saturation> 85% with FiO2 of 30%), and full feeding tolerance so that oral Fe can be administrated.

Also we excluded all neonates with acquired or congenital infections, intracranial hemorrhage, vomiting or diarrhea, malabsorption, another types of anemia (ABO incompatibility and G6PD deficiency), or life threatening anomalies. The sample size was calculated according to 80% powers and 95% confidence. Seven neonates were allocated for each group. A written consent form was taken from both parents.

The results in the 2 groups were compared and analyzed with SPSS software (Version11.5, Chicago, IL, USA) and t-paired and repeated measurement tests.

## Results

Thirty subjects were enrolled in the study. The average birth weight of neonates in the first group was 1632±313 g and in the second group was 1826±399 g.The weight gaining pattern in the two groups did not have any significant difference (p=0.151). According to Table 1 in every group, Hb level at birth and day 28, a significant difference was observed (p<0.001). A significant difference in Hb level between the 2 groups was noticed (p<0.001) (Table 1).

**Table 1:** Mean of Hb at the birth day and 28th day.

	**Subcutaneous rEPO Mean±SD **	**Oral rEPO Mean ±SD **	**All**
Birth day	14.1 ± 1.7	2.5 ± 13.2	2.6±14.2
28th day	12.5 ± 1.7	1.9 ± 9.7	2.3±11.1

The patients receiving blood were excluded. A significant difference (p<0.001) regarding Hb level and repeated measurement test, the level of HCT at the birth day and day 28 showed a significant difference (p<0.001) regarding the 2 groups (Table 2).

**Table 2:** Mean of HCT at the birth day and 28th day.

	**Subcutaneous rEPO Mean±SD**	**Oral rEPO Mean±SD**	**All**
Birth day	45.2 ± 6.5	39 ± 6.4	42.1±7
28th day	38.8 ± 5.1	29.6 ± 5.9	34.2±7.2

After excluding the patients who received blood , the results showed a significant differences again and after omitting the recipients of blood, no significant difference was observed (p=0.412) (Table 3).

**Table 3:** Mean of weight gaining in 2 groups in difference days.

**Birthday**	**Oral rEPO Mean±SD**	**Subcutaneous rEPO Mean±SD**	**All**	***P *value**
3^th^ Day	186±1140	163±1245	180±1193	0.110
7^th^ Day	237±1201	190±1304	217±1253	0.211
10^th^ Day	212±1248	283±1370	249±1302	0.202
14^th^ Day	196±1276	331±1472	282±1370	0.071
17^th^ Day	297±1355	349±1584	338±1465	0.071
21^th^ Day	309±1428	360±1680	344±1580	0.170
24^th^ Day	296±1540	372±1759	347±1644	0.121
28^th^ Day	313±1632	399±1826	365±1729	0.151

## Discussion

In this study, EPO administration either orally or subcutaneously prevented prematurity anemia in the 4th week, but better results were obtained in the subcutaneous EPO group. In Ohls’s study, treatment with oral and IV EPO was effective in preventing the decrease of Hb and HCT to the level of anemia of prematurity (<7 mg/dl) so that neither groups had a Hb less than 7 g/dL in the 4th week after birth.[12]

In the oral EPO group, average of Hb in 4th week was 9.7±1.9 g/dL in the IV EPO group, which shows a significant statistical difference (p<0.001). However we can say that IV EPO was more effective than the oral form. If we notice that in the oral rhEPO group, the Hb level was less than the IV group, we can say that" It is probable that oral rhEPO is as effective as IV rhEPO since the fall in Hb in the two groups in 4th week did not have much difference.

In our study, similar amount of blood was withdrawn from the two groups before and during the investigation period, but the need for transfusion in the oral rhEPO group was more. We performed blood transfusion on 3 neonates of the oral rhEPO group (20%) and one neonate in the subcutaneous rhEPO group (6%).

On the other hand, according to various studies showing that 50% of neonates had birth weight less than 500 g, there was a need for multiple transfusions of packed cells. So oral and subcutaneous rhEPO were effective in decreasing the need for blood transfusion.[3] In a prospective study in which 6 neonates did not receive rhEPO, it was shown that rhEPO could not increase erythropoiesis.[1]

Ballin et al. showed that rhEPO mixed with milk and administrated orally could increase erythropoesis in premature neonates. Serum erythropoietin level and reticulocytes could increase the level of ferritin which shows that Fe stores were depleted in order to be used in Hb synthesis.[1]

Similar results were obtained in our study as in the oral rhEPO group, the Hb level at birth and 4th weeks after treatment showed a significant difference. Of course, serum erythropoietin level and reticulocyte count were not obtained in our study. Britten et al. reported that in 6 neonates who received 1000 u/kg oral rhEPO for 10 days, high levels of serum rhEPO two hours after administrating the drug were recognized. Although oral rhEPO increased erythropoesis, but the need to packed cell transfusion did not decrease.[3] These results matches with our results since in our study 3 out of 15 neonates needed packed cell transfusion. A retrospective study was conducted by Ledbetter in order to study the effect of rhEPO on human GI system.[9]

Neonates having a 1250 g birth weight and admitted to their NICU between the years 1993 and 1997, and 260 neonates received rhEPO and another 233 neonates were allocated as a control group. The incidence of NEC following treatment decreased (4.61% compared to 10.76%, p<0.05).

Gestational age in treated neonates was less than control group (26.8±2.1 compared to 27.6 ±2.9 weeks, p<0.05). Birth weight, APGAR of 1st minute and gender prevalence were not different in 2 groups. It was shown that in premature neonates with 500-1250 g birth weight, the incidence of NEC in the rhEPO receiving group was less. Feeding intolerance (residue increase) and vomiting were not observed in neonates receiving oral rhEPO.9 Although in our study the effects on GI system were not investigated but in this study orally EPO prevented prematurity anemia. In this investigation, it was shown that rhEPO administration (400 u/kg) either orally or subcutaneously in the 4th week of birth prevented the incidence of prematurity anemia. However, better results were obtained in the subcutaneous EPO group.
